# Supplementary figures and images for: Characteristics of spatiotemporal distribution of HIV-1 Gag-containing complexes on the dorsal membrane tracking with live confocal imaging
Source: PLoS One. 2025 Dec 29;20(12):e0339593. doi: 10.1371/journal.pone.0339593 (PMC12747361; doi:10.1371/journal.pone.0339593)

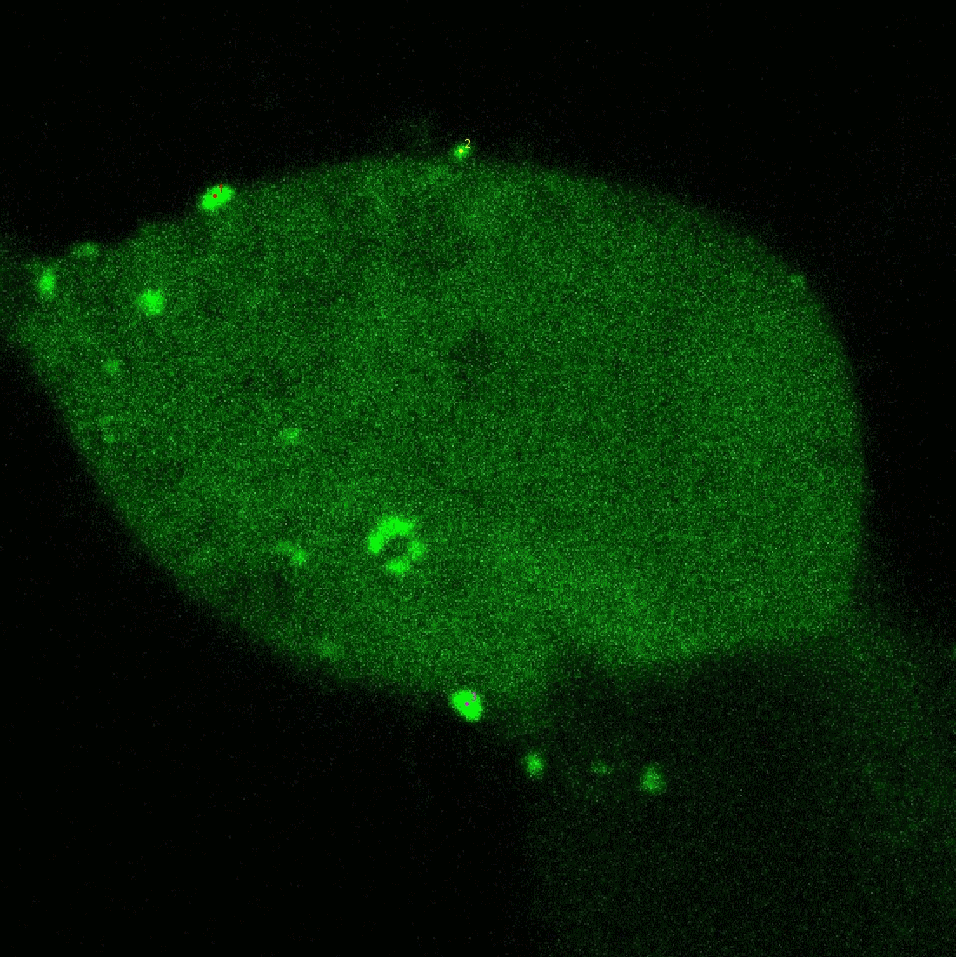

Supplement: S1 Movie — (GIF) [file pone.0339593.s001.gif]

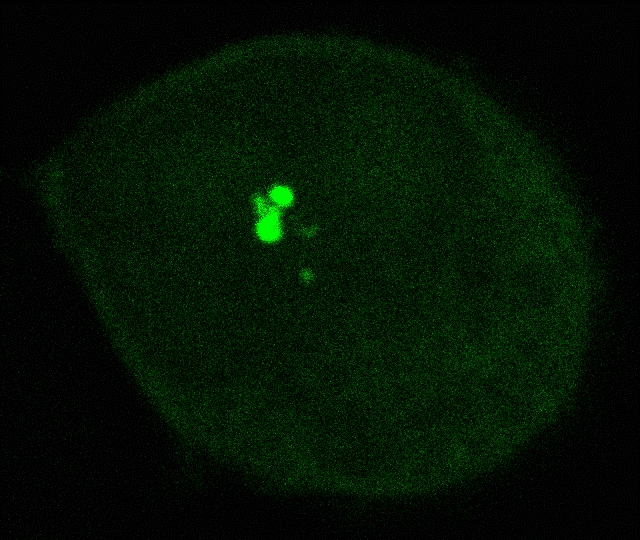

Supplement: S2 Movie — (GIF) [file pone.0339593.s002.gif]

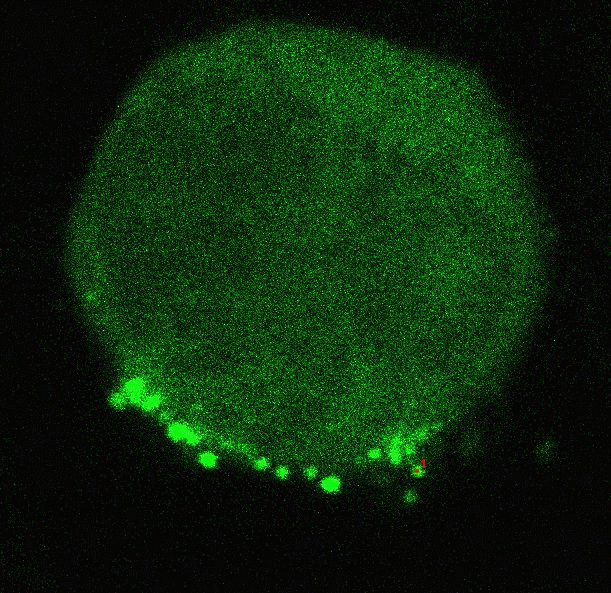

Supplement: S3 Movie — (GIF) [file pone.0339593.s003.gif]

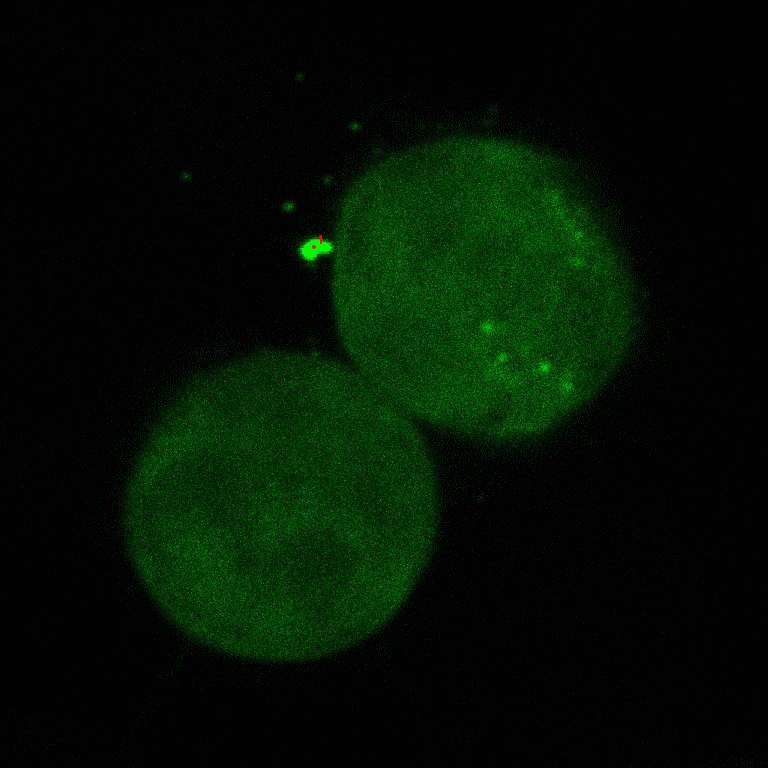

Supplement: S4 Movie — (GIF) [file pone.0339593.s004.gif]

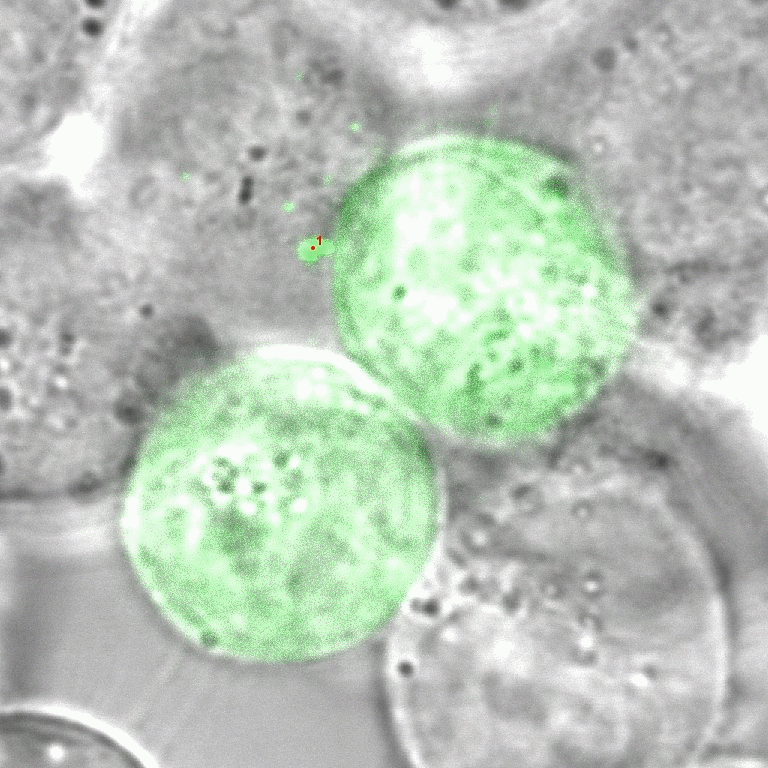

Supplement: S5 Movie — (GIF) [file pone.0339593.s005.gif]

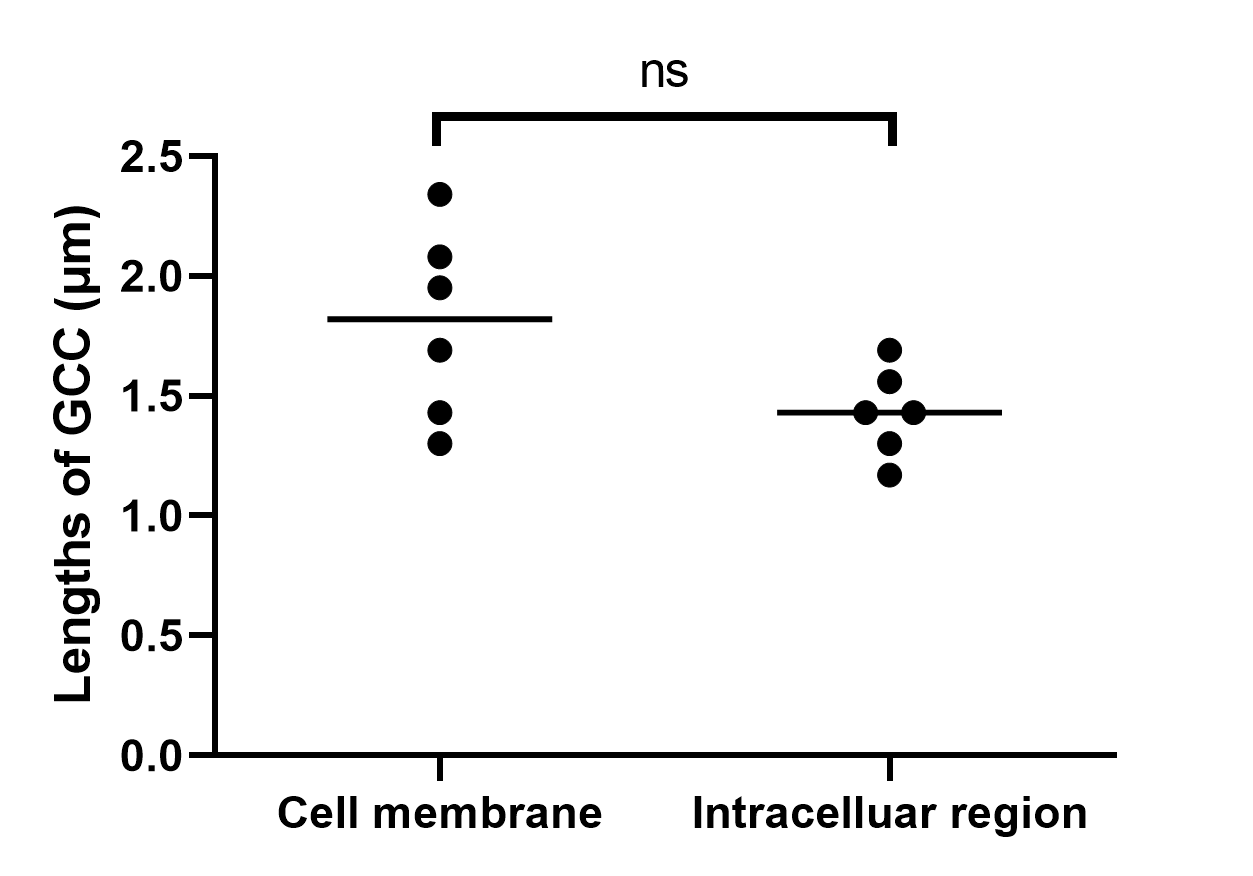

Supplement: S1 Fig — (TIF) [file pone.0339593.s006.tif]

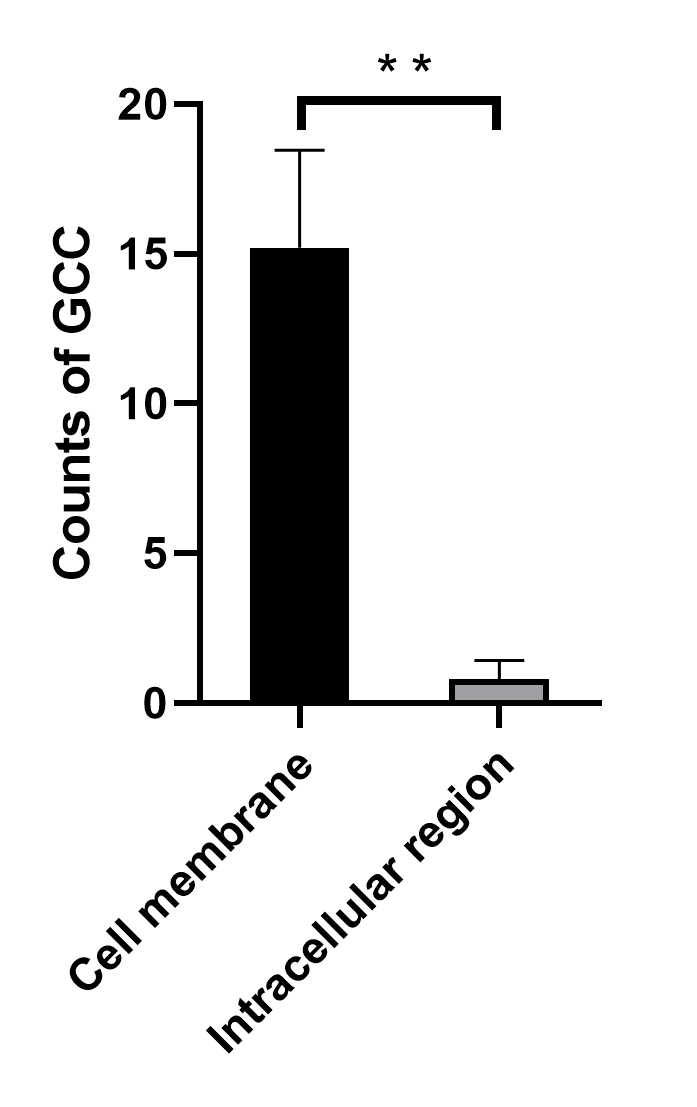

Supplement: S2 Fig — (TIF) [file pone.0339593.s007.tif]
