# Supplementary material for: Characteristics of spatiotemporal distribution of HIV-1 Gag-containing complexes on the dorsal membrane tracking with live confocal imaging
Source: PLoS One. 2025 Dec 29;20(12):e0339593. doi: 10.1371/journal.pone.0339593 (PMC12747361; doi:10.1371/journal.pone.0339593)
Supplement: Attachment — Submitted filename: Response to Reviewers-20251208.docx [file pone.0339593.s010.docx]

Dear Editor and Reviewers:

We appreciate your valuable comments on our manuscript. These comments have provided substantial guidance for our research. We have thoroughly examined the comments and made appropriate corrections. The revised sections in the paper are highlighted in red. Below, we present the main corrections made to the paper and our responses to the editor and reviewers’ comments.

**Editor：**

The extreme brevity of the Methods and Materials sections makes replication of the experiments impossible and thus inappropriate for PLOS One.

**Q1.	This section does not explain how much plasmid was used.**

For transfection, HEK293T cells (1.5 × 10⁵) were seeded into 35 mm confocal dishes (NEST). On the day of transfection, the cells had reached 50% to 60% confluency. HEK293T cells were transfected with pEGFP-N3 vectors or pEGFP-N3-Gag vectors (3 μg/well).

**Q2.	It is not clear if the plasmid contains p6 and Env, whether non-tagged Gag was used？**

The plasmid lacks the p6 and Env genes, and untagged Gag was not employed. To dynamically monitor the spatiotemporal distribution of HIV-1 Gag-containing complexes, HEK293T cells were transfected with either pEGFP-N3 vectors or pEGFP-N3-Gag vectors (3 μg/well), and then subjected to detection using laser confocal microscopy.

The cDNA of HIV p55 was amplified by polymerase chain reaction (PCR) from the pLP1 vector (Invitrogen) and then cloned into the pEGFP-N3 vector using the restriction enzymes Xho I and BamH I. The coding sequence of HIV p55 (Gag) spans bases 1355-2857.

**Q3.	The number of replicates?**

Each experiment was replicated at least three times. Relevant contents have been supplemented in the chapter of statistical analysis of materials and methods.

**Q4.	The number of stacks?**

1) In Figure3, the number of stacks is 60. This content has been described in the results section of the manuscript.

( “At the same time, the 60th scanned image (away from the glass surface) revealed a more dispersed distribution of Gag-EGFP proteins, with some appearing as small circular dots (Fig 3E2).”)

2) In Figure4, the number of stacks is 25. This content has been described in the results section of the manuscript.

( “To further elucidate the spatial distribution characteristics of Gag-EGFP proteins in the cytoplasm, LCM images were continuously captured using xyz scanning mode (layers 1–25).”)

**Q5.	The length of the movies?**

**Movie S1.gif:** The 5-second video file has a playback speed of 2 frames per second.

**Movie S2.gif:** The 9-second video file has a playback speed of 2 frames per second.

**Movie S3.gif:** The 14.5-second video file has a playback speed of 2 frames per second.

**Movie S4.gif:** The 9-second video file has a playback speed of 2 frames per second.

**Movie S5.gif:** The 9-second video file has a playback speed of 2 frames per second.

The above content has been described in the supplementary results section of the manuscript.

**Q6.	The statistical methods?**

**Statistical analysis**

Statistical analyses were conducted using an unpaired Student’s t-test in GraphPad Prism 9 (San Diego, USA). Each experiment was replicated at least three times, and the data are presented as means ± standard deviation (SD). Differences were considered statistically significant when the p-value was less than 0.05.

**Q7.	There should be a statistical analysis performed on the data of Fig S1 and given its importance it merits to be moved to the main text.**

In this study, the distribution regions of Gag proteins on the cell membrane or within the cell ranged approximately from 1 to 2 µm (Fig S1). Nevertheless, there was no significant statistical difference in the length of the Gag proteins between the two groups (P > 0.05).

The above content has been described in the discussion section of the manuscript. (Page 8 of the manuscript, in red font)

**Q8.	Figures A, B and C in the response to the reviewers are really important and should be in the main text (indicating the proper statistical analysis).**

In this study, the distribution regions of Gag proteins on the cell membrane or within the cell ranged approximately from 1 to 2 µm (Fig S1). Nevertheless, there was no significant statistical difference in the length of the Gag proteins between the two groups (P > 0.05). In Fig S2, we found that the distribution of GCC on the dorsal cell membrane significantly exceeded its distribution inside the cell (P < 0.01). At the same time, the quantity of GCC on the cell membrane or inside the cell exhibits certain variations at different observation levels (data not shown).

**Figures A:** Fig S2

**Figures B:** data not shown

**Figures C:** Fig S1

The content has been described in the discussion section of the manuscript. (Page 8 of the manuscript, in red font)

**Reviewers**

**Q1:** Only one more minor suggestion: in the discussion part, the authors write: "In this study, the lengths of the thin, elongated, cord-like Gag proteins distributed on the cell membrane or inside the cell are approximately 1-2 µm". This is ambiguous, I think the author means the region of distribution for Gag proteins range is approximately 1-2µm, instead of the protein itself. No matter the monomer Gag alone, or the immature Gag/Gag-pol lattice are far shorter/thinner than 1-2µm (size for a whole HIV-1 virion is usually less than 150nm).

Revise as follows:

In this study, the distribution regions of Gag proteins on the cell membrane or within the cell ranged approximately from 1 to 2 µm (Fig S1).

The content has been described in the discussion section of the manuscript. (Page 8 of the manuscript, in red font)
